# Feasibility of Licensed Vocational Nurses Using a CDS App to Communicate Signs and Symptoms of a UTI

**DOI:** 10.1155/nrp/8574727

**Published:** 2025-03-07

**Authors:** Alyce S. Ashcraft, Donna C. Owen, Kyle Johnson, Huaxin Song

**Affiliations:** , School of Nursing, Texas Tech University Health Sciences Center, 3601 4 Street, Stop 6264, Lubbock, Texas 79430, USA

## Abstract

Urinary tract infections (UTIs) occurring in nursing home (NH) residents are poorly assessed and challenging to treat. The emergence of clinical decision support (CDS) technology provides an opportunity for improved diagnosis and treatment of UTIs in the NH. The purpose of this study was to report findings examining the feasibility of licensed vocational nurses (LVNs) using a CDS algorithm designed to directly guide assessment of a standardized NH resident experiencing symptoms of a potential UTI in a simulation setting at a university in the Southern United States. A structured observational design was used. A sample of ten practicing nurses used an algorithm developed by the authors from published UTI assessment and practice-driven criteria. Data collection was framed using (a) UTI-guided assessment tool, (b) analysis of LVN behavior and verbal interaction with the algorithm, and (c) postsimulation interviews about the algorithm and nurse–resident interactions. Results showed LVNs found the algorithm easy or very easy to use, their behaviors demonstrated high levels of engagement with the simulation while using the algorithm, and interviews supported the positive value LVNs placed on using an algorithmic approach for UTI assessment. The algorithm we developed fills a gap in the current approaches to diagnosing a UTI in the NH by focusing on the role of the LVN in data collection in relation to assessment of the resident.

## 1. Introduction

Urinary tract infections (UTIs) are one of the most common infections in long-term care, yet they are poorly assessed and challenging to treat [1]. Despite the nationally driven priority for antibiotic stewardship in the United States, few approaches have been successful in changing prescribing practices. The negative impact of developing antibiotic resistance in nursing home (NH) residents [2] emphasizes the need to ensure licensed vocational nurses (LVNs) working in the NH are fully capable of detecting the signs and symptoms of UTIs. A meta-analysis noted a 45% prevalence of UTIs in hospitalized patients [3], but UTI prevalence has been harder to characterize in NH residents [4]. UTIs in the NH differ from UTIs occurring in acute care settings because NH residents are predominantly over 65, live in the NH for longer periods of time, and have multiple comorbidities, including higher levels of cognitive impairment and weakened immune systems [5]. The signs and symptoms of a UTI are often subtle, especially in NH residents with dementia, who may be unable to verbally communicate and make their needs known. LVNs, also known as licensed practical nurses, are the caregivers in the NH responsible for assessing resident infections. LVNs are entry level healthcare providers who practice under the supervision of registered nurses (RNs) or physicians. Together, RNs and LVNs make sure each resident's plan of care is being followed and their needs are being met. LVNs typically have one year of training, a directed scope of practice, and require state Board of Nursing licensure [6].

LVNs use broadly written NH protocols that do not focus on disease-specific evidence-based assessment criteria. Similarly, such protocols do not direct how to efficiently collate data gathered or how to communicate assessment findings with primary care providers (PCPs) attempting to make treatment decisions. Ashraf et al. [1] refer to “entrenched practice patterns” of NH staff that interfere with diagnosis and prevent appropriate antibiotic use.

To address needed changes in the practice patterns, we see the emergence of technology to offer new approaches to improve decision making in the diagnosis and treatment of UTIs in the NH. New et al. [7] used an algorithm to identify the signs and symptoms associated with a suspected UTI and incorporated a paper-based clinical decision support (CDS) tool into real-time care with improvements in appropriate antibiotic prescribing. Other UTI CDS tools have been developed but have not been examined in clinical trials [8, 9]. These tools are designed to be used by PCPs and NH nurses [10] and range in focus from testing algorithms for feasibility [6], collecting existing data from the EHR [11], and testing CDS for directing appropriate urine culture collection [12]. These varied approaches to UTI CDS speak to the need for current electronic platforms to be further developed in conjunction with the encoding of clinical guidelines and guided questions related to particular signs and symptoms of common diagnoses in high-risk populations and inclusion of PCPs and nursing staff [13].

The algorithm we developed fills a gap in the current approaches to diagnosing a UTI in the NH. Our algorithm relies on the familiarity of the LVN with the usual protocols for assessment of the resident's UTI-related physical and behavior changes directly or from an EHR, the collection of a urine sample, and collating the information for communication to the PCP. Effective communication of the resident's signs and symptoms from nurse to PCP is important for diagnostic accuracy and resident management of infections. In the NH, communication starts with the LVN feeling empowered and valued for their input regarding resident status. This requires continuing education about common resident conditions and, if available, frequent guidance regarding electronic communication methodologies to support more effective and efficient care. The purpose of this paper is to report findings examining the feasibility of LVNs using CDS to guide collection of information with a standardized NH resident experiencing symptoms of a suspected UTI in a simulation setting at a university in the Southern United States.

## 2. Methods

### 2.1. Study Design

A structured observational design was used. Research team members used behavioral checklists while viewing the video-taped experience of LVNs interacting with a nursing home resident in a high-fidelity simulation [14, 15]. Quantitative and qualitative description was used in an accredited simulation setting with a high-fidelity manikin.

### 2.2. Study Setting

A simulation setting was selected for this study because it afforded the opportunity to examine CDS from the perspective of using technology for assessing a NH resident with a UTI. A simulation was designed and facilitated according to the International Nursing Association for Clinical Simulation and Learning (INACSL) Standards of Best Practice: Simulation^SM^ [16]. A single scenario portraying a cognitively impaired NH resident with a UTI included a standardized caregiver in the role of an unlicensed nursing assistant for the LVN to ask questions about the usual behaviors of the resident. At the onset of data collection, the LVN was told that the resident had a change in condition, to meet the nursing assistant in the room, and use an iPad with the UTI assessment questions to evaluate the resident. Prior to entering the room, the LVN was oriented to the iPad.

### 2.3. Sample

After obtaining university IRB approval, participants were recruited from the university's ambulatory clinics to obtain a convenience sample of ten practicing LVNs. Ambulatory care LVNs with clinical experience using an iPad for routine patient data collection were selected. We chose these LVNs because we wanted to examine the algorithm driving the assessment of the UTI versus familiarity with and use of an iPad [17]. A minimum sample size of 10 was desired to ensure ability to evaluate the feasibility goals [18].

### 2.4. Materials and Measures

We were testing the underlying script, the algorithm, for a CDS app. The script was placed in a survey format in order to examine the feasibility of using UTI assessment questions on an iPad. We were determining if this evidence-based script could guide LVNs in their assessment of the resident. The script was designed to obtain LVN responses recorded within the Qualtrics software using a combination of (a) guided assessment data entry by the LVN as directed by published UTI assessment criteria [1, 19–21] (b) LVN behavior and verbal interaction during video recording of the simulated assessment, and (c) audio-recording transcripts of the post-simulation interview about the feasibility of collecting data via iPad. Questions addressed both the functionality of using an iPad while assessing a NH resident as well as the order and content directed by the CDS algorithm. Using the CDS algorithm on the iPad required direct physical assessment, verbal questioning of the resident and nursing assistant, and abstraction of information from a medical record in paper chart format.

#### 2.4.1. Guided UTI Assessment

The investigator developed guided assessment questions consisting of 28 items presented in a fixed sequence requiring a forced response. Each of the 28 items was created to determine if the resident was exhibiting signs and symptoms characteristic of a UTI. Typical items included the presence of a urinary catheter, symptoms such as dehydration, urinary incontinence or frequency, and location or type of pain. Additional items asked the LVN to locate specific lab values and vital signs from a bedside medical record. After completion of the Guided UTI Assessment, overall ease of use of the iPad was rated by participants on a 10-point scale, where 10 indicated very difficult to use. The left column of Table 1 depicts the open-ended questions asked to understand participant reasoning when using the iPad, comparing their usual experience when assessing a UTI against our directed assessment questions.

#### 2.4.2. LVN Behavior and Verbal Interaction

An observation checklist was developed for video recordings of LVN and resident verbal and behavioral interaction (see Table 2). A checklist was created for coders to use while viewing videos and tabulating results across multiple coders. The checklist consisted of 12 items and included the frequency of LVN interaction and eye contact with the resident and potential interference of the iPad with person-to-person interaction. Interactions were defined as initiation of conversation through speech, touch, or eye contact by the LVN with the NH resident. We watched for use of the iPad to determine if it would pull participant focus away from interacting with the resident. The interaction was noted to end when the LVN stopped speaking and making contact with the resident. The checklist was scored by recording the occurrence of each item on the checklist and tallying the total number of times each item on the checklist occurred. This created a total frequency score for each item on the checklist. Investigators developed a codebook of decisions to facilitate consistent scoring. Three investigators independently coded each video and came to consensus.

#### 2.4.3. Post-Simulation Interview

One investigator met with each participant to review select points in the video recordings to clarify participant thoughts during the simulation. Seven open-ended UTI Assessment Questions (Table 1) were asked of each participant. Answers from the UTI Assessment Questions were used to determine feasibility of collecting data using the Guided UTI Assessment via iPad in concordance with UTI assessments done in other settings.

### 2.5. Data Analysis

The three sources of data in this study were (a) the data collected within a Qualtrics survey as subjects used the iPad to enter answers to questions posed in the order specified by the UTI Guided Assessment algorithm, (b) video recording of LVN assessing the simulated patient, and (c) interview transcripts of investigator talking with the LVN after completing the UTI Assessment. As we analyzed each data source, we focused on enhancing the trustworthiness of the data and making choices in how we approached the data analysis [22].

#### 2.5.1. Survey Data Analysis

We were interested in determining if the algorithm correctly captured the data being entered by the participant in the order specified.

Guided assessment was tracked in Qualtrics by data entered into the iPad by the LVN. To enhance trustworthiness of the data, Qualtrics forms for each trial were examined to determine completeness of data. The examination was done independently by two investigators who then discussed findings and looked for discrepancies between their reviews. Information on the Qualtrics forms provided duration of the overall UTI Guided Assessment for the simulated patient and time spent considering and responding to each item. Each question with the assessment was tracked to see if it was completed and what information was entered, selected, or left blank.

#### 2.5.2. Video Data Analysis

LVN behavior and verbal interaction were video recorded, and recordings analyzed using focused and selective behavioral observation [15]. Interviews included investigator–participant watching of recordings to enable debriefing of participants to help us understand their thinking about specific moments and actions during their resident assessment. All video recordings were viewed by one investigator (DC). Next the investigator (DC) chose one video, identifying each new interaction between the LVN and the resident, noting the time stamp for each, and taking notes about coding decisions. This resulted in the creation of a list of coding rules. The coding rules addressed how to determine the beginning and end of an interaction, recording of the time stamp, and how to determine the presence of eye contact and physical touch. The same investigator then viewed the same video, applying the coding rules, and compared the first and second reviews of the video. A second reviewer (AA) used the coding rules to independently review the same video. Slight modifications to the coding rules were made to add clarity. The two reviewers then independently coded the remaining videos. A third reviewer (KJ) then used the final coding rules to independently code all the videos. The coding of the videos by all three coders was compared. There was agreement among the three coders on the identification and frequency of interactions. Investigators noted when eye contact or physical contact with the resident was initiated and ended by the LVN. Each episode of eye or physical contact was tallied by each investigator. For each video, a mean score was determined for eye contact and physical contact across the three reviewers.

#### 2.5.3. Interview Transcript Data

Post-simulation interview data came from audio-recorded interviews with each participant. Transcripts were reviewed against recordings. NVivo was used to manage the analysis of the transcripts. Interview questions are included in Table 1. Content analysis of the interview data guided examination of answers to interview questions and for overall comments about the CDS algorithm [23]. Answers were coded as positive or negative, and specific concerns were labeled and grouped together. For example, interview analysis focused on listing individual participant answers to our question about what they expected the CDS algorithm to make them include in their assessment of a resident with a UTI. These lists of expectations were then labeled as positive, negative, and coded by labeling expectations. Expectations were noted to be similar or different from their usual assessment approach. Coding of expectation focused on placing comments into Missing from Algorithm and Similar to Algorithm categories. We purposefully looked for comments suggesting modification of the algorithm and created a category for Changes to Algorithm.

Trustworthiness was attended to by using the four criteria proposed by Guba [24] including credibility, dependability, confirmability, and transferability. Credibility was addressed by collecting referential materials and expertise of investigators to consider along with investigator observation of the simulation. We also used peer debriefing by interviewing each participant immediately after the simulation while looking at the audiovisual recording together. Analysis of each interview was begun shortly after the interview in order to consider the use of additional questions or probes in subsequent interviews. We also had 3 of the investigators viewing and coding the interaction behavior the LVN with the simulated patient. The investigator brought expertise in using high-fidelity simulation, debriefing in combination with audiovisual recordings. This enabled us to clarify the meaning of select behaviors of the LVN. Dependability was supported by the use of a standardized protocol for the conduct of the simulation and debriefing in combination with the audiovisual. All comments from participants and using a comparative strategy of across comments strengthened the uniformity of our approach. Confirmability was addressed through the use of triangulation of data from three sources to enhance our understanding of the behavior and perspective of the LVN throughout the simulation. Participant experiences conducting assessments and using other CDS devices were integrated with comments about specific components or working features of the CDS data in the analysis. Transferability was addressed to some extent by achieving data saturation. We were not seeing additional challenges brought up by study participants. Interview data were enhanced by the inclusion of all comments from participants and using a comparative strategy across comments. The use of single coder and group discussion and consensus of the analysis strengthened the credibility and confirmability of the data [25].

### 2.6. Ethical Considerations

Institutional Review Board approval was obtained prior to data collection. Participants were provided with a written and verbal description of the study. They were told about what activities they would be expected to take part in, including the nature of the simulation and the video and audio recording of their assessment. All participants provided written consent. A gift card of $10 was provided to each participant at the completion of the scenario. Data were maintained on university encrypted/password protected servers.

## 3. Results

### 3.1. Sample Demographics

The sample consisted of 9 females and 1 male, ranging in age from 23 to 52 (Mdn = 39 and 42 years) who had between 1 and 30 years of experience as an LVN (Mdn = 11 and 13 years). Six identified as non-Hispanic, three as Hispanic, and one no response. Seven identified as white, one African American, and two Asian American. Two previously worked in NHs and all currently worked with older adults.

### 3.2. Guided UTI Assessment

Time required to complete the assessment on the iPad averaged 11.20 (SD = 4.67) minutes. All questions were answered by each participant. Mean score for overall ease of use for the CDS algorithm was 1.0 (SD = 1.2; range 0–4) (rating scale 0 = very easy, 10 = very difficult). Positive and negative comments about ease of use and suggested changes to the algorithm are presented in Table 1. LVNs engaged with the resident's nursing assistant, moving quickly through items on the iPad using physical contact with the resident as needed. Participants found it difficult to locate laboratory results in the medical record and did not know normal values. There was also limited knowledge of drugs used for treatment of dementia. Participants wanted to add a question about urine odor or color to the algorithm. Both of these are signs of dehydration [26] and are not diagnostically useful for confirming a UTI. Once completed, the information was submitted using a secure link to a mock PCP. This algorithmic approach was uncomfortable for some LVNs because they were used to saying more with less structure, “I just tell them everything I know about the resident.”

### 3.3. LVN Behavior and Verbal Interaction

The observation checklist is presented in Table 2. Verbal interaction frequency mean was 17.9 (SD = ±7.2), and eye contact mean was 10.6 (SD = ±4.1). Participants made physical or eye contact during 67% of interactions; six participants deviated from questions to ascertain urine odor and color. LVNs positioned the iPad by placing it on a table or holding it so they could see the questions while making eye contact with the resident.

### 3.4. Post-Simulation Interview

Answers to UTI assessment questions were primarily positive, noting using an iPad was familiar and the questions were expected for assessing a UTI (see Table 1). Negative comments included locating laboratory results in the medical record and not understanding the value of lab results without a stated normal range. Drug names presented in the scenario were not recognized by all participants. Several participants wanted to add a question about urine odor or color. One participant suggested that we add free formed text boxes to allow unrestricted number and length of comments to physicians.

## 4. Discussion

The purpose of this study was to examine the feasibility of LVNs using an algorithm guiding collection of information with a standardized NH resident experiencing symptoms of a suspected UTI in a simulation setting. Our results are framed around using (a) a Guided UTI Assessment Tool, (b) analyzing LVN behavior and verbal interaction with the tool and simulated resident, and (c) post-simulation interview findings about the tool and their interactions.

There are many benefits to the use of technology in the NH environment, including streamlined workflow and accessible information at the point of care [16]. It is important to recognize that clinicians may be skeptical and nonadherent with training and procedures and unsure of how the devices may be maintained in the long term [17]. In our study, we were able to demonstrate LVNs were able to use an algorithm to guide collection of data determined to be necessary based on CDS criteria for UTI screening. Our participants were familiar with using an iPad for data collection, focusing more on content that was required to answer the questions. The LVNs easily worked through the algorithm in a simulated NH setting. Our next step will be to take this algorithm to the actual clinical setting where workflow will be a major factor in the acceptance of LVN usage.

Simulations show expectations of actual interactions in the clinical setting. We expected LVN behavior and verbal interaction to be incorporated by the LVNs as a part of their resident assessment despite it being a simulation. A study by Jin et al. [27] used more sophisticated strategies for observing nonverbal interaction and found the simulation setting was adequate for fully engaging nurse–resident interaction. Our study also confirmed the nurse–resident engagement as the LVNs demonstrated physical touch and eye contact when assessing a resident with a UTI. Our findings suggest the suitability of creating further supportive technology for LVN assessment of NH residents.

Understanding the impact of using a Guided UTI Assessment on the LVN, both in terms of their thinking and the behaviors they exhibited during the assessment, and interaction with the NH resident are key to looking for potential changes to the algorithm. The post-simulation interviews highlighted problems the LVN identified while using the iPad and completing the UTI assessment. Nurse–resident interaction was positively compared with and without the algorithm. In contrast, Harvey and Powell [17] reported the device itself could be a barrier to nurse–resident interaction. Improvements to using the algorithm as suggested by the LVNs were to include additional information about residents at their fingertips. This is just the exact point of technology to adapt to the needs of the user. Further iterations of the algorithm and ultimately the full development of a CDS app should include sufficient flexibility to accommodate the end user needs.

### 4.1. Limitations

This feasibility study was conducted to determine the possibility of using an algorithmic approach to assess a UTI on an iPad. Understanding the full capacity of the CDS algorithm was limited by a small convenience sample, testing in a simulated versus live setting, using participants not currently working in a NH setting, and not having the PCP perspective of the CDS algorithm coinciding with LVN use. While conducting this feasibility study might have been enhanced by using nurses in the NH setting, we were able to focus on the interaction of the LVN with the algorithm and provide insight into the acceptability of using an iPad when assessing NH residents.

## 5. Conclusions and Recommendations

The purpose of this study was to examine the feasibility of LVNs using an evidence-based algorithm guiding collection of information with a standardized NH resident experiencing symptoms of a suspected UTI in a simulation setting. The algorithm on the iPad was easy to use and navigate. The algorithm directing the order of the UTI assessment made sense to the LVNs. To enhance usability, access to medication information directly from the iPad was wanted. Point-of-care evidence-based UTI assessment by LVNs can be directed through use of a CDS algorithm.

Future recommendations are to expand this type of algorithmic approach for LVNs in the NH. This device would be more useful if it included expanding the number of resident problems on a device and testing the device in multiple settings. The LVNs followed the structured questions and completed the requested information; however, including more ways to add “just in time learning” with medication information and lab values would be helpful to the nurses. Emerging technologies need to be considered to update delivery devices to keep the information accessible and useable. Examining feasibility of this algorithmic approach sets the stage for supporting the education and practice of LVNs in long-term care.

## Figures and Tables

**Table 1:** Interview questions and select examples.

Questions	Respondent comments (selected)
*Ease of CDS use*	
1. How easy was it to use the assessment? (rated 0–10) 2. How easy is it to use the assessment? (comments) 3. How easy was it to navigate through the questions on the iPad? 4. How hard was it to use the iPad when talking with the resident or nurse aide?	• 0 to 10 scale (0 = very easy and 10 = very difficult) • Very easy, I had no problem, I'm used to using an iPad in the clinic with patients • No, it was pretty fluid. I mean, I was able to scroll through everything. Maybe make the fonts a little bit bigger on some of the ranges, the values for some that can't really read it clearly. I guess make it bold in some cases... • At first, I had a hard time figuring out where to stand but then it was easy

*UTI assessment questions*	
1. Were the questions you were being directed to ask similar to ones you already use with residents with a possible UTI? 2. Were the choices you were given what you expected? 3. Was the information you were collecting what you were expecting to find out from a resident with a possible UTI? 4. Was the wording of questions and directions easy to follow? 5. Did the directions help you know what to say to the resident? 6. Was anything missing from the questions or directions that you would usually find out about when collecting information from a resident you suspected had a UTI? 7. Was anything missing that you would usually report to a physician about a resident you suspected had a UTI?	• Yes, I already ask questions like this. I didn't know the drug [Aricept] • No. We just do the assessment depending on what we found. Most of the time when patients with dementia, they couldn't really tell me, but this patient really could. We would go by sometimes the smell of odor or maybe a little confusion or just them not being themselves, like maybe run down, you know, something's not right • It was good having a nurse aide there as well. But overall, pretty good options where you could do the assessment I thought • I thought it was good that it asked about blood in the urine, because that's a big thing • I guess on the lab work, that's where I kind of messed up. I was looking for the exact value, like what we normally do in the clinic setting—it would help to have the normal [range] right there • It was easy to look down and quickly know what to say. It was like having a suggestion or prompt. Yes, I wanted to tell the physician more about the resident. I usually tell them what is wrong with the patient and other problems they have. The odor and color of the urine is missing

**Table 2:** Observation checklist.

Start video and log the following:
Event (UTI)
Observer initials
Date of event
Start time
LVN initials
Nursing assistant initials
Listen for LVN comments; make a tally for each person she/he talks to watch for LVN position in relation to the resident (how close to the resident, next to the nursing assistant or across from her)
Watch for eye contact
a. Create a tally of eye contact with the resident
b. Create a tally of eye contact with the nursing assistant
c. When checking the iPad does she/he talk with the resident with or without eye contact
Watch for touching
a. Who does she/he touch
b. Where does she/he touch
c. Create a tally of touch with the resident
d. Create a tally of eye contact with the nursing assistant
e. Does she/he touch resident as part of assessment Watch for use of iPad

## Data Availability

The data used in this study are available upon request from Dr. Alyce Ashcraft.
